# The Role of the Akt Signaling Pathway in Sjögren’s Syndrome

**DOI:** 10.31138/mjr.34.1.113

**Published:** 2023-03-31

**Authors:** Efstathia K. Kapsogeorgou, Ioanna E. Stergiou, Loukas Chatzis, Michael Voulgarelis, Panayiotis G. Vlachoyiannopoulos

**Affiliations:** Department of Pathophysiology, School of Medicine, National and Kapodistrian University of Athens, Greece

**Keywords:** Akt signalling pathway, Sjögren’s syndrome, infiltrating mononuclear cells, minor salivary glands, non-Hodgkin lymphoma, salivary gland epithelial cells

## Abstract

Primary Sjögren’s syndrome (pSS) is a chronic autoimmune disorder with diverse clinical picture and high prevalence of B-cell non-Hodgkin lymphoma (NHL), that possibly raises from the chronic activation of B-cells. The mechanisms underlying the development of neoplasia in pSS remain elusive. Activated Akt/mTOR pathway is a uniform finding in cancer, whereas its significance in haematologic malignancies is highlighted by the plethora of inhibitors with promising therapeutic efficacy. PI3K-Akt activation has been involved in the TLR3-induced apoptosis of cultured salivary gland epithelial cells (SGECs), whereas upregulated expression of the phosphorylated ribosomal S6 protein (pS6), an end-result of PI3K signalling, has been detected in the infiltrating T and B lymphocytes at the MSG lesions of pSS patients; nevertheless, without specifying if this was mediated by the Akt/mTOR or Ras/ERK pathways. To this end, the role of Akt/mTOR pathway in pSS and associated lymphomagenesis, will be investigated by the immunohistochemical detection of the entire and phosphorylated protein forms of Akt kinase and two of its substrates, namely the FoxO1 transcription factor and the proline-rich Akt substrate of 40-kDa (PRAS40) in MSGs of pSS patients with variable histological and clinical phenotype, as well as sicca-complaining controls. Subsequently, the role of this pathway will be evaluated in in-vitro inhibition experiments, studying the effect of specific inhibitors in the phenotype, function, and interaction of SGECs and B cells. The current proposal is expected to promote the understanding of pSS pathogenesis, enlighten the mechanisms underlying related lymphomagenesis and possible therapeutic targets.

## BACKGROUND / INTRODUCTION

Primary Sjögren’s syndrome (pSS) is a chronic autoimmune disorder with a diverse clinical picture that ranges from a mild, limited to exocrine glands, to severe/life-threatening, multi-systemic disease with serious implications for life quality and productivity and development of B-cell non-Hodgkin lymphoma (NHL) in 6-10% of patients.^1^ In the majority of pSS patients, NHL starts in the exocrine glands, such as the salivary glands, which are the major target of pSS autoimmune responses. NHL represents the major adverse outcome of the disease, influencing both morbidity and mortality.^1–6^ The high frequency of transformation to lymphoid malignancy in pSS among autoimmune rheumatic diseases^7,8^ and the accessibility of the affected organ, the minor salivary glands (MSG), render pSS an ideal model for the study of lymphomagenesis associated with autoimmune diseases and inflammation. The mechanisms underlying the development of neoplasia in pSS remain elusive. The existence of several clinical, laboratory and histological features present at diagnosis, including salivary gland enlargement (SGE), purpura, vasculitis, leukopenia, cryoglobulinemia, hypocomplementemia, rheumatoid factor, the severity of histopathologic MSG infiltrates, their organization into germinal centres (GC) and the infiltration by certain cell types, such as macrophages, in the pSS patients who are at high-risk to develop NHL in the future^3,9–15^ suggest that it is a chronic, multi-step process. Generally, it is considered that lymphomagenesis in pSS is a multistep process that arises from the chronic, continuous, antigen-driven B-cell stimulation resulting in the ineffective control of IgV gene recombination, chromosomal translocations, activation of proto-oncogenes, inactivation of tumour-suppressor genes, and ultimately to malignant transformation.^14,16^ The cellular source of the antigenic stimulation of B cells and the type of antigens are elusive. The organization of MSG infiltrates in ectopic germinal centres (GC) is considered critical for the activation of autoreactive B cells and the development of MALT lymphomas.^15,17,18^ Furthermore, salivary gland epithelial cells (SGECs) that are the key regulators of the pSS autoimmune responses in MSGs,^19,20^ have been shown to drive the differentiation of B cells, in a similar manner to that observed in MSG lesions,^19,21^ suggesting that they may be involved in the chronic activation of B cells and lymphomagenesis. The molecular pathways underlying the lymphomagenesis in pSS are under study.

Akt is a phosphoinositide-dependent serine/threonine kinase that regulates cell cycle, metabolism, proliferation, cell survival, apoptosis, growth, and angiogenesis in response to extracellular signals provided by growth factors, cytokines and other stimuli.^22^ Therefore, it is directly related to cellular senescence, proliferation, apoptosis, and cancer. The PI3K/Akt/mTOR pathway operates as follows: the external signal, eg, growth factor, binds to the receptor tyrosine kinases (RTKs) which activate the PI3 Kinase (PI3K) that phosphorylates phosphatidylinositol biphosphate (PIP2) to D3-phosphorylated phosphoinositides (PIP3). These bind to both phosphoinositide-dependent kinase 1 (PDK1) and Akt protein, recruit Akt protein at the cell membrane, allowing PDK1 to reach and phosphorylate threonine-308 (T308) of Akt, leading to its partial activation. Then, Akt is phosphorylated at serine-473 (S473) by the mammalian target of Rapamycyin (mTOR) Complex-2 (mTORC2) resulting in full Akt activation. Activated Akt translocates to the cytoplasm and nucleus, where it phosphorylates a number of substrates implicated in the afore-mentioned cellular procedures, including critical regulators of cell growth, survival and apoptosis, such as the FoxO1 transcription factor, p53 oncogene, the proline-rich Akt substrate of 40 kDa (PRAS40), which is a component of mTORC1.^23–28^

Activated PI3K/Akt/mTOR pathway is a uniform finding in cancer, including hematologic malignancies,^29,30^ whereas its significance in oncogenesis is highlighted by the plethora of inhibitors (dual PI3K–mTOR inhibitors, PI3K inhibitors, Akt inhibitors and mTOR inhibitors) that are in clinical development for the treatment of cancer, with promising results in hematologic malignancies.^30–32^

Little is known for the role of Akt/mTOR pathway in the pathogenesis of pSS. PI3K-Akt activation has been involved in the TLR3-induced apoptosis of cultured salivary gland epithelial cells (SGECs),^33^ whereas upregulated expression of the phosphorylated ribosomal S6 protein (pS6), an end-result of PI3K signalling, has been detected in the infiltrating T and B lymphocytes at the MSG lesions of pSS patients^34,35^; nevertheless, without specifying if this was mediated by the Akt/mTOR or Ras/ERK pathways. This data suggest that Akt/mTOR pathway may be implicated in the pathogenesis of pSS and related lymphomagenesis.

## AIM OF THE STUDY

The current proposal aims to study the implication of the Akt/mTOR pathway in the pathogenesis of pSS. This approach is expected to reveal new pathogenetic pathways with therapeutic potential and to promote the understanding of the mechanisms underlying the lymphomagenesis in pSS. The latter is critical for the development novel therapeutic approaches and possibly for the containment of lymphoma development.

## RESEARCH PLAN – METHODS

The activation of AKT and its signalling pathway will be examined by studying immunohistochemically the expression of both the entire and activated (phosphorylated) forms of the major molecules comprising the Akt/mTOR pathway, namely Akt and its substrates FoxO1 and PRAS40, in MSGs from pSS patients (low-risk for lymphoma, prelymphoma, and lymphoma), as well as sicca controls (patients who had been subjected to MSG biopsy due to sicca symptoms, but had negative biopsy and did not express any autoantibodies). Furthermore, depending on the findings in MSGs, the expression of the afore-mentioned molecules will be also analysed in SGECs and B cells from pSS patients and sicca controls by western blotting. The analysis of the entire and phosphorylated protein expression of each molecule will be performed by commercially available antibodies [entire Akt: clone C67E7 (Cell Signalling Technology), Akt phosphorylated at T308 (phosphoAkt-T308, Origene), Akt phosphorylated at S473 (phosphoAkt-S473, clone EP2109Y, Abcam), PRAS40 (clone D23C7, Cell Signalling Technology), phosphorylated PRAS40 at T246 (phosphoPRAS40-T246, polyclonal, Abcam), FoxO1 (clone D29H4, Cell Signalling Technology) and FoxO1 phosphorylated at S319 (phosphoFoxO1-S319, polyclonal, Abcam)]. In addition, the cell types expressing these molecules will be identified immunohistochemically using specific markers, including cytokeratins for epithelial cells, CD3 and CD20 for T and B lymphocytes, respectively, and CD21 for follicular dendritic cells.

In case of differential expression of Akt pathway related molecules in pSS patients at high risk to develop lymphoma (as we hypothesise based on preliminary experiments), the role of this pathway will be studied in functional in vitro inhibition experiments, where SGECs and B cells from pSS patients will be cultured in the presence of specific inhibitors, such as LY294002, followed by phenotypic analysis using flow cytometry, western blotting and evaluation of cytokine production by commercially available ELISA kits. Furthermore, the effect of the inhibition of AKT pathway in the interaction of SGECs and B cells and in the SGEC-driven B cell activation will be investigated by the addition of specific inhibitors in the co-culture systems and phenotypic analysis of B cells by flow cytometry, as well as by evaluating the cytokine production in culture supernatants by commercially available ELISAs.

The study has been approved by the Ethics Committee of School of Medicine, NKUA, Greece (Protocol-No.: 489), whereas our preliminary results presented below support the feasibility of the project.

### Preliminary results that feed into our project

The expression of phosphorylated AKT at S473 has been examined immunohistochemically in MSG tissues obtained from a) two pSS patients without NHL, b) one pSS patient two years before MALT-NHL diagnosis (pre-lymphoma), c) the same pSS patient on NHL diagnosis and d) two sicca-controls. Expression of the fully activated AKT was detected in the ductal epithelia and the infiltrating mononuclear cells at the MSGs obtained from the patient who developed MALT (before and on NHL onset), but not in the tissues from the patient without lymphoma or the sicca-controls (data not shown). These findings suggest that Akt/mTOR pathway is activated in the pSS patients who are going to develop or have lymphoma and is implicated in pSS-related lymphomagenesis.

## IMPACT OF THE STUDY

The study is expected to enlighten the pathogenetic mechanisms underlying pSS pathogenesis and related lymphomagenesis. Furthermore, considering the existence of therapeutic regimens targeting Akt/mTOR pathway, the findings of the present study may justify the evidence-based therapeutic administration of these agents, for the treatment and/or prevention of the pSS-related NHL. This is of high importance, since up to date, the use of expensive biological treatments, such as B cell depletion therapy, has poor results in pSS treatment and related lymphoma.
